# Electrochemical Surface Modification of Laser Cladded Ni-Based Single Crystal Superalloy in NaNO_3_ Solution

**DOI:** 10.3390/ma18214967

**Published:** 2025-10-30

**Authors:** Jingbo Liu, Yongxin Liu, Xianqi Meng, Linfeng Tang, Xiaowei Lei, Nan Wang

**Affiliations:** 1 Guiyang AECC Power Investment Casting Co., Ltd., Guiyang 550014, China; orangesky@163.com; 2 School of Physical Science and Technology, Northwestern Polytechnical University, Xi’an 710072, China; nwpulyx@mail.nwpu.edu.cn (Y.L.); xiaowei_lei@nwpu.edu.cn (X.L.); 3 Ningbo Boway Alloy High-Tech Wire Co., Ltd., Ningbo 315135, China; felix.meng@bedra.cn; 4 Capital Aerospace Machinery Co., Ltd., Beijing 100076, China; tanglinfeng301@163.com

**Keywords:** Ni-based single crystal superalloy, laser metal deposition, electrochemical dissolution

## Abstract

Since mechanical processing can introduce stress in the sample, electrochemical dissolution has been utilized to attain shape accuracy in certain materials. However, this technique is rarely applied to laser-repaired Ni-based single-crystal superalloys. In this work, the transpassive dissolution behaviors of an additive manufacturing-repaired Ni-based single crystal superalloy in a 10% NaNO_3_ solution were investigated by comparison with the substrate. A significant disparity in dissolution rates was found between the dendritic and interdendritic regions of the substrate, resulting in a rough surface. Conversely, the dissolution of the dendritic and interdendritic regions in the cladding structure occurred nearly simultaneously, leading to a high-quality, smooth surface. This behavior was attributed to the differences in phase dissolution preferences between the substrate and the cladding structure. It indicates that electrochemical dissolution is a promising method for achieving shape accuracy in laser-clad Ni-based single-crystal superalloys.

## 1. Introduction

Ni-based single crystal (SX) superalloys are mainly applied to manufacture the gas turbine engine blades [1,2]. With the increase in service temperature, the enhanced solid solution of refractory elements between γ/γ′ phases further improves the high-temperature strength of Ni-based SX superalloys. Nevertheless, their performance will deteriorate due to crack failure in harsh service environments [3]. To extend the lifespan of the defective SX components, laser additive manufacturing (LAM) has emerged as a cost-effective repair approach [4,5]. Ni-based SX superalloys are repaired via laser repairing through epitaxial growth, a metallurgical bonding mechanism that ensures microstructural integrity in the repaired region. Previous research successfully implemented the laser repairing process for the DD6 alloy and analyzed the formation mechanism of stray grains through theoretical calculations [6,7]. After the repair process, mechanical processing is still necessary to attain high dimensional and shape accuracy. However, conventional processing methods come with certain drawbacks, like recrystallization and tool wear [8,9]. For this reason, some new processing methods should be developed.

Electrochemical dissolution has demonstrated the capability to reduce surface hardness while improving shape accuracy and has been applied to the Ni-based SX superalloys [10,11]. It should be noted that the previous investigations mainly focused on the SX substrate after standard heat treatment. The study focuses on the microstructure of as-cast Ni-based single crystal superalloys, electrochemical parameters, and the influence of corrosive solutions on their electrochemical behavior. During LAM, rapid solidification occurs, and it significantly influences the microstructure, resulting in different sizes and shapes of the γ (Ni solid solution) and γ′ (Ni_3_Al) phases, as well as dendrite morphology [11,12,13,14]. Moreover, during the laser cladding process, the characteristics of epitaxially grown microstructures are inconsistent between the vertical build direction and the direction horizontal to the build direction [15]. Guo et al. [16] investigated the electrochemical behavior of Inconel 718 fabricated by laser solid forming in 10 wt.% NaNO_3_ and found no significant difference in corrosion resistance between the vertical and horizontal fabrication directions. Liu et al. [17] investigated the electrochemical dissolution behavior of directionally solidified Ni-based SX superalloys in different electrolytes. They found that differences in the dendritic and interdendritic regions of the solidified microstructure led to variations in the density of the passive film, which, in turn, resulted in differential dissolution rates within the microstructure. Up to date, the electrochemical dissolution behaviors of the γ/γ′ phases and the dendritic/interdendritic regions in the LAM-repaired SX superalloy remain unclear.

In this study, we will investigate the electrochemical dissolution behavior of the LAM-repaired Ni-based SX superalloy and compare it with the substrate in a NaNO_3_ solution. The underlying mechanism of different dissolution behaviors in the LAM-repaired part and the substrate will be determined.

## 2. Materials and Methods

In the laser cladding experiment, a directionally solidified (001) SX superalloy DD6 was used as the substrate for epitaxial growth of the subsequent cladding microstructure. Both the substrate and the powder used in laser cladding are composed of (wt%) is 4.3Cr -9Co-2Mo-8W-7.5Ta-2Re-0.5Nb-5.6Al-0.1Hf, with the balance Ni. For the cladding, the laser power was set to 750 W and the scanning speed to 50 mm/s. During the experiment, the sample was placed in a transparent glass chamber filled with argon gas to prevent oxidation of the molten pool. After the experiments, park-erosion cutting was performed to obtain cladding samples parallel and perpendicular to the build direction. For ease of subsequent description, the vertical section and horizontal section are abbreviated as VS and HS. The cladding layer was polished by using 2000-grit SiC sandpaper (Keyan, Guangzhou, China) and cleaned, followed by the electrochemical dissolution experiments. Electrochemical measurements were performed using a traditional three-electrode setup, including a KCl-saturated Ag/AgCl reference electrode, a platinum counter electrode, and the specimen serving as the working electrode in NaNO_3_ solutions (99.5%, Guanghua, China, Guangdong). To characterize the microstructures, A Zeiss Axio A1 optical microscope (Zeiss, Oberkochen, Germany) and an FEI Verios G4 UC scanning electron microscope (SEM; ThermoFisher Scientific, Hillsboro, OR, USA) with an energy-dispersive spectroscopy (EDS) detector were used. Characterization of electrochemical dissolution morphology of cladding microstructure and substrate using 3D microscopy (Hirox-100, Takarazuka, Japan). The 3D topography is reconstructed by acquiring the height and precision information of the alloy surface using the confocal principle.

## 3. Results and Discussions

**Figure 1:**
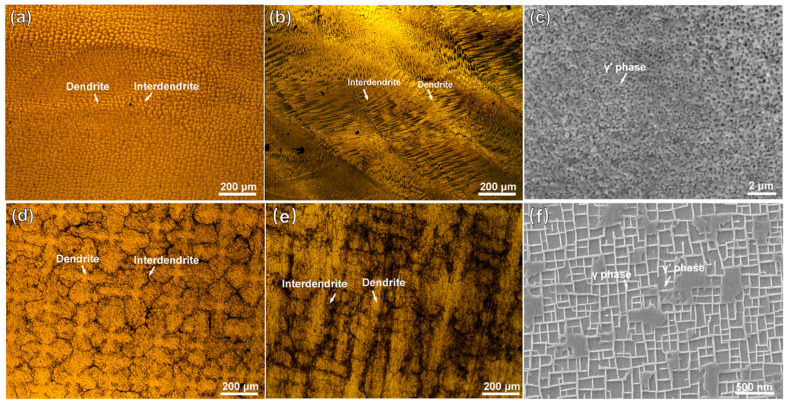
OM and SEM images of the microstructures of the DD6 superalloy: (**a**,**b**) representing OM images of the HS (Horizontal Section) and VS (Vertical Section) planes of the cladding microstructure, respectively; (**c**) SEM image of the γ/γ′ phases in the cladding sample; (**d**,**e**) representing OM images of the HS (Horizontal Section) and VS (Vertical Section) planes of the substrate, respectively; (**f**) SEM image of the γ/γ′ phases in the cladding sample. (HS-horizontal section, VS-vertical section).

Figure 1 shows the microstructures of the substrate and the cladding layer on different sections. In Figure 1a, the manufactured structure of the cladding layer shows fine and dense dendrite features. On the VS surface of the cladding layer, as shown in Figure 1b, fine dendrites grow upward along the substrate orientation. A magnified SEM image in Figure 1c reveals abundant nanoscale particles of γ′ (Ni_3_Al) phases. As shown in Figure 1d, the substrate exhibits a typical dendritic/interdendritic morphology. In Figure 1e, the microstructure image of the substrate’s VS surface clearly shows coarse dendrite trunks within the matrix, where the cuboidal γ′ phase and grid-like γ phase are presented in Figure 1f. Compared to the substrate, which underwent standard heat treatment, the accelerated cooling rate during the LAM process results in the precipitation of finer γ′ phases from the γ phase in the cladding layer.

**Figure 2:**
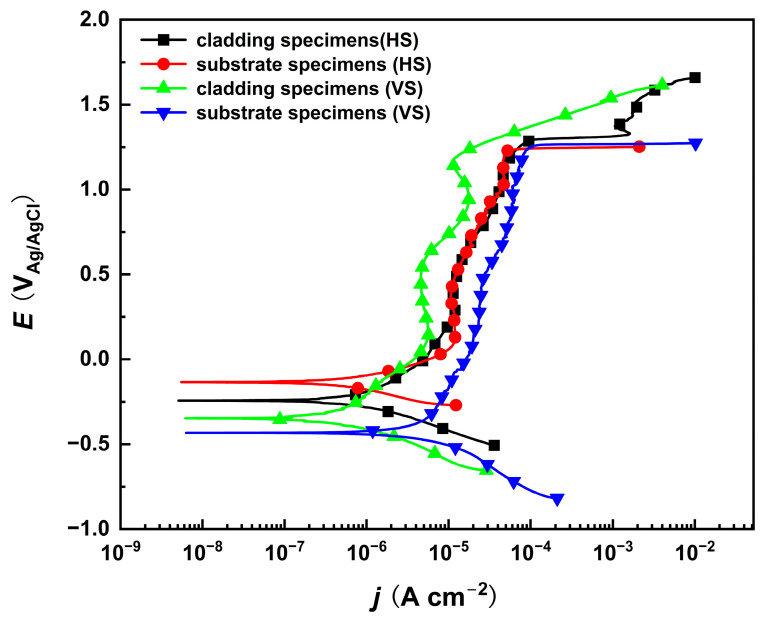
The potentiodynamic polarization curves of the cladded sample and the substrate on the HS and VS surfaces in 10% NaNO_3_ solution. (HS-horizontal section, VS-vertical section).

Potentiodynamic polarization tests of the substrate and cladding layer on different sections were performed in a 10% NaNO_3_ solution, as shown in Figure 2. The corrosion potentials of both the substrate and the cladding layer on the HS surface are higher than those on their respective VS surfaces. This indicates that the HS surface has a preferential tendency to corrode. In the passive region where the current density changes very slowly within a certain potential range, the corrosion current density of the cladding layer is slightly lower than that of the substrate, implying that a compact passive film is produced on the cladding specimen. Although the corrosion rate differs in the passive region, the polarization curves indicate that the passivation breakdown potential remains consistent. This indicates that both the cladding layer and the substrate exhibit identical transpassive dissolution tendencies on their HS and VS surfaces near a specific transpassive potential, which is favorable for subsequent anodic dissolution experiments. However, due to the significant microstructural differences between the cladding layer and the substrate, further characterization and analysis of the dissolution morphology are still required.

**Figure 3:**
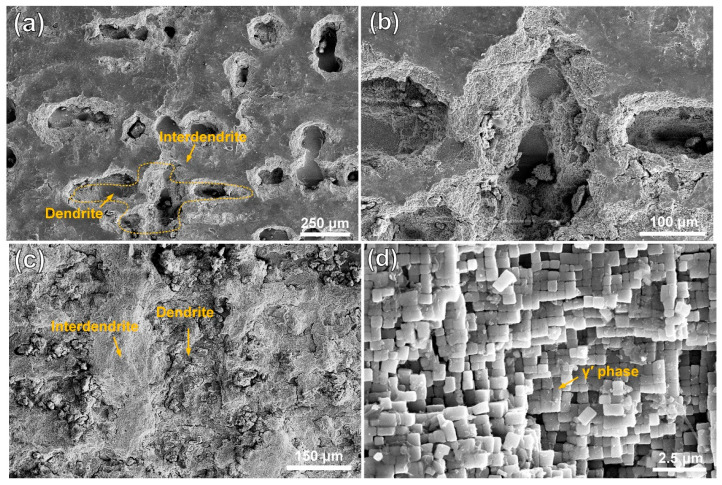
Dissolution morphology of the substrate in 10% NaNO_3_ solution, characterized by SEM secondary electron detection. (**a**) the dendritic and interdendritic corrosion morphologies on the HS surface of the substrate; (**b**) magnified view of the orange region in (**a**). (**c**) the dendritic and interdendritic corrosion morphologies on the VS surface of the substrate; (**d**) high-resolution characterization results of the γ/γ′ phase in the orange region of (**c**). (HS-horizontal section, VS-vertical section).

**Figure 4:**
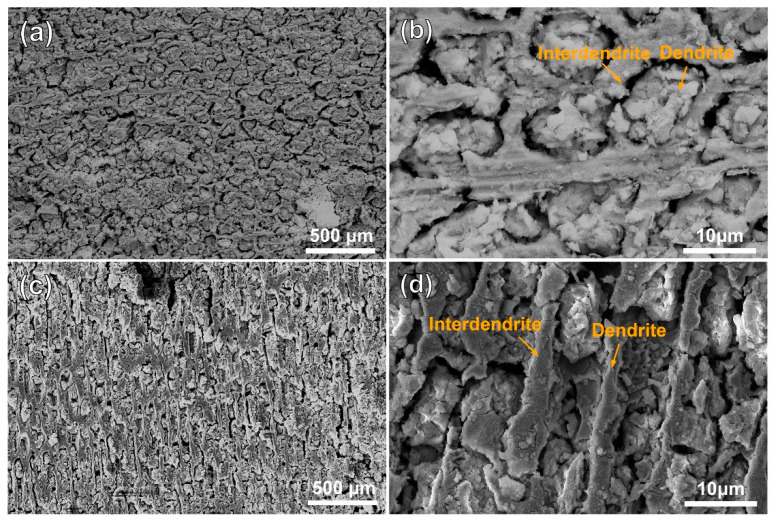
Dissolution morphology of the cladding specimens in 10% NaNO_3_ solution characterized by SEM secondary electron detection. (**a**) the dendritic and interdendritic corrosion morphologies on the HS surface of the cladding specimens; (**b**) magnified view of the orange region in (**a**). (**c**) the dendritic and interdendritic corrosion morphologies on the VS surface of the cladding specimens; (**d**) magnified view of the orange region in (**c**). (HS-horizontal section, VS-vertical section).

Figure 3 and Figure 4 show the morphologies of the substrate and the cladding specimen after transpassive dissolution at 2.2 V_Ag/AgCl_ in NaNO_3_ solution. In Figure 3a,b, the SEM images of the HS surface of the substrate reveal the formation of cross-shaped pits, resulting from preferential corrosion of the dendritic regions. Slight dissolution occurs in the interdendritic regions, leading to a corrosion morphology with height differences compared to the deeply dissolved dendritic regions. The corrosion morphology of the substrate’s VS surface, as depicted in Figure 3c, shows that dendrite trunk dissolution occurred similarly while the interdendritic regions remained undissolved, forming vertical channels. Characterization of the dissolution morphology of the γ/γ′ phases shows that preferential dissolution of γ phases occurred on the entire surface of the substrate, and the retained γ′ phase formed columnar dissolution features shown in Figure 3d. The significant difference in dissolution rates of the substrate leads to an increase in surface roughness following electrochemical dissolution. In contrast, Figure 4a,b shows that the surfaces of the cladding sample on both the HS and VS sides are uniformly flat after dissolution. The high-magnification images in Figure 4c,d reveal that the dendrite trunks underwent significantly more dissolution than the interdendritic regions, and this dissolution trend is consistent with that observed in the substrate.

**Figure 5:**
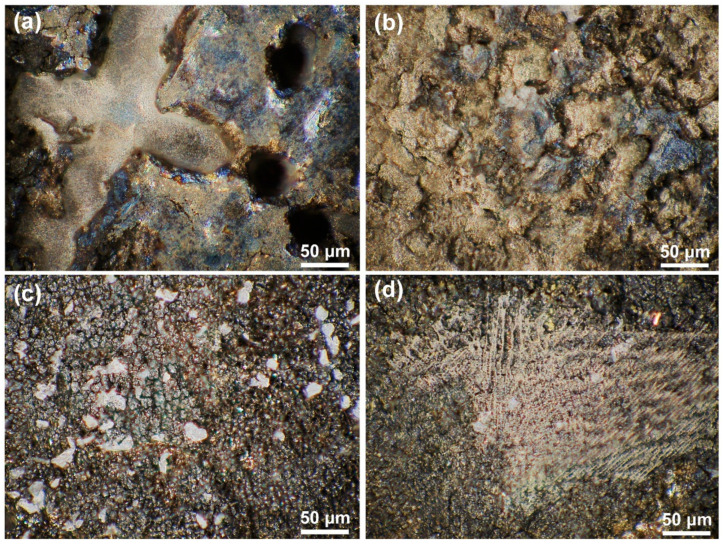
The OM morphologies of the cladding specimens and the substrate on the HS and VS surfaces after dissolution. (**a**) the HS surface of the substrate; (**b**) the VS surface of the substrate; (**c**) the HS surface of the cladding specimens; (**d**) the VS surface of the cladding specimens. (HS-horizontal section, VS-vertical section).

**Figure 6:**
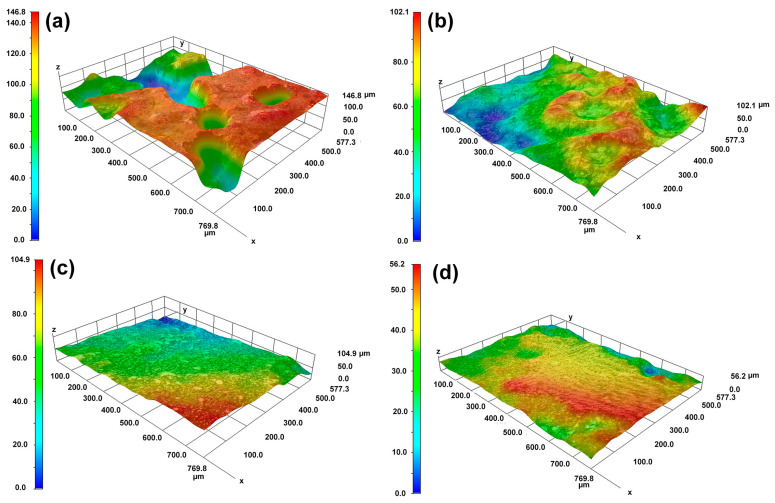
The 3D morphologies of the cladding specimens and the substrate on the HS and VS surfaces after dissolution. (**a**) the HS surface of the substrate; (**b**) the VS surface of the substrate; (**c**) the HS surface of the cladding specimens; (**d**) the VS surface of the cladding specimens. (HS-horizontal section, VS-vertical section).

The OM morphological image in Figure 5 and the 3D topography in Figure 6 further characterize the differences in dissolution between the substrate and the cladding microstructure on different surfaces. Figure 5a,b reveals distinct topographic undulations in the dendrite and interdendritic regions after the substrate dissolution. The OM microscope results in Figure 5c,d indicate that the cladding microstructure has a flat surface after dissolution. The maximum height difference in Figure 6a is 120 μm for the HS surface of the substrate and 90 μm in Figure 6b for the VS surface of the substrate. In contrast, although the observation region for the HS surface of the cladding specimen is inclined a little bit, the results presented in Figure 6c show that the maximum height difference is less than 40 μm. The maximum height difference on the VS surface of the cladding sample is less than 30 μm, as shown in Figure 6d. The 3D topography analysis distinctly indicates that the surface quality has been greatly improved in the mixed solution. The 3D topography analysis clearly shows that, under the same dissolution conditions, the surface quality of the cladding sample is superior to that of the substrate.

**Figure 7:**
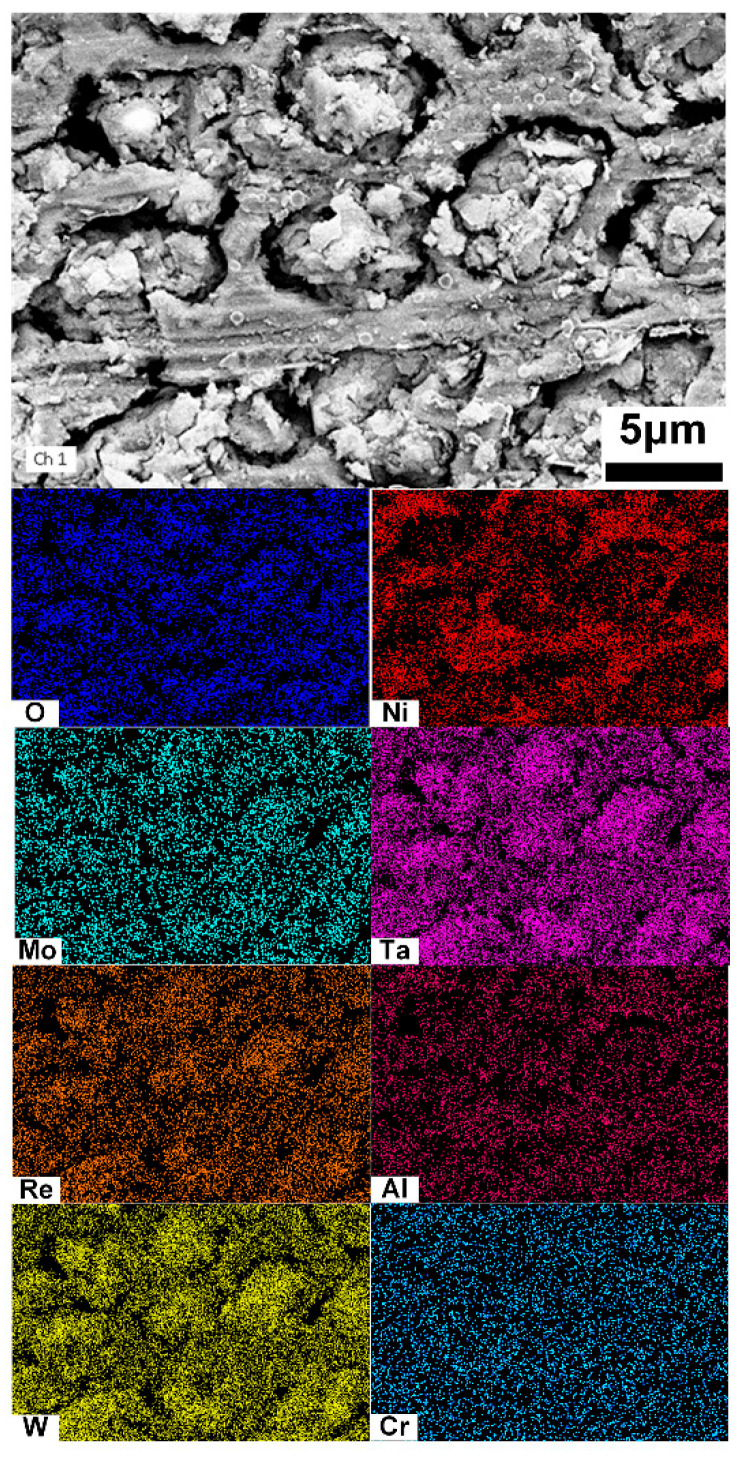
EDS analysis of the cladding layer after electrochemical dissolution.

The distributions of the dissolved surface in the cladding layer were examined using SEM–EDS, as shown in Figure 7. The oxides of Ta and W are enriched in the dissolved dendritic regions, while Ni oxides are predominantly concentrated in the interdendritic regions.

Now we discuss why the substrate and the cladding layer exhibit different behaviours of electrochemical dissolution. In the substrate, as shown in our previous study [17], the dendritic region exhibits a continuous Cr-rich γ phase with a higher volume fraction compared to the interdendritic regions. Chromium (Cr) exhibits a strong tendency to hydrolyze, leading to an increase in acidity in the corrosive environment around the γ′ phase. This oxidation, combined with high anodic potential, promotes the active dissolution of the γ matrix. The dissolution of the γ phase in the dendritic region creates preferential pathways for the electrolyte to penetrate, further facilitating the reaction process between ions and the phase surface.

Due to differences in the sequence of directional solidification and solute distribution, the γ′ phases in the interdendritic regions are larger and γ discontinuous compared to those in the dendritic regions, which interrupts the dissolution path and results in a lower dissolution rate. In the cladding layer, the γ′ phase size (50 nm) is several times smaller than that of the substrate (700 nm), which diminishes the difference in dissolution rates between the dendritic and interdendritic regions. Smaller phases a blso enhance the uniform distribution of stable oxides of Cr, Mo, and W elements within the passive film, further improving its compactness. This effect is corroborated by the results presented in Figure 2, which show lower passivation current density in the smaller cladding microstructure. Equation (1) represents the dissolution process of the film in an acidic environment based on the Point Defect Model (PDM) [18].

(1) $${MO}_{\chi /2}+\chi H^{+}\to M^{\delta +}(aq)+\frac{\chi }{2}H_{2}O+(\delta -\chi )e^{\prime }$$

where m is the metal atom, $M^{\chi +}$ is the metal cation in solution, $M_{M}$ stands for the metal cation on the metal sublattice of the passive film, $O_{O}$ represents the oxygen anion on the oxygen sublattice of the passive film, and $MO_{\chi /2}$ is the stoichiometric passive film oxide.

The more uniform passive film of the cladding microstructure, compared to the DD6 matrix, maintains the same dissolution rate at each surface site during electrochemical dissolution, leading to uniform dissolution of the surface layer. Consequently, these regions dissolve simultaneously at a similar rate, resulting in a flatter dissolved surface. This indicates that taking NaNO_3_ solution could potentially result in shape accuracy in the repaired components. Certainly, the future large-scale application of electrochemical dissolution for Ni-based SX superalloys will face certain technical limitations. The mass production of SX turbine blades requires industrial-grade electrodes, where the Ohmic drop leads to non-uniform current distribution. The choice of electrolytes significantly impacts the depth of surface corrosion and the composition of oxides formed. Therefore, further detailed parameter optimization is essential for the industrial implementation of this technology.

## 4. Conclusions

The transpassive dissolution behavior of an additive manufacturing-repaired DD6 single-crystal superalloy in 10% NaNO_3_ solution was studied and compared with that of the substrate. Compared to the substrate, the cladding layer is more susceptible to transpassive dissolution. Meanwhile, the dissolution of the dendritic and interdendritic regions within the cladding structure occurs nearly simultaneously, resulting in a high-quality, smooth dissolved surface. This is potential for application to the shape accuracy of the repaired SX components in the future.

## Data Availability

The original contributions presented in this study are included in the article. Further inquiries can be directed to the corresponding author.
